# Expression and Functional Roles of Angiopoietin-2 in Skeletal Muscles

**DOI:** 10.1371/journal.pone.0022882

**Published:** 2011-07-29

**Authors:** Mahroo Mofarrahi, Sabah N. A. Hussain

**Affiliations:** Critical Care and Respiratory Divisions, Royal Victoria Hospital, McGill University Health Centre, and Meakins-Christie Laboratories, Department of Medicine, McGill University, Montreal, Quebec, Canada; University of Queensland, Australia

## Abstract

**Background:**

Angiopoietin-1 (ANGPT1) and angiopoietin-2 (ANGPT2) are angiogenesis factors that modulate endothelial cell differentiation, survival and stability. Recent studies have suggested that skeletal muscle precursor cells constitutively express ANGPT1 and adhere to recombinant ANGPT1 and ANGPT2 proteins. It remains unclear whether or not they also express ANGPT2, or if ANGPT2 regulates the myogenesis program of muscle precursors. In this study, ANGPT2 regulatory factors and the effects of ANGPT2 on proliferation, migration, differentiation and survival were identified in cultured primary skeletal myoblasts. The cellular networks involved in the actions of ANGPT2 on skeletal muscle cells were also analyzed.

**Methodology/Principal Findings:**

Primary skeletal myoblasts were isolated from human and mouse muscles. Skeletal myoblast survival, proliferation, migration and differentiation were measured in-vitro in response to recombinant ANGPT2 protein and to enhanced ANGPT2 expression delivered with adenoviruses. Real-time PCR and ELISA measurements revealed the presence of constitutive ANGPT2 expression in these cells. This expression increased significantly during myoblast differentiation into myotubes. In human myoblasts, ANGPT2 expression was induced by H_2_O_2_, but not by TNFα, IL1β or IL6. ANGPT2 significantly enhanced myoblast differentiation and survival, but had no influence on proliferation or migration. ANGPT2-induced survival was mediated through activation of the ERK1/2 and PI-3 kinase/AKT pathways. Microarray analysis revealed that ANGPT2 upregulates genes involved in the regulation of cell survival, protein synthesis, glucose uptake and free fatty oxidation.

**Conclusion/Significance:**

Skeletal muscle precursors constitutively express ANGPT2 and this expression is upregulated during differentiation into myotubes. Reactive oxygen species exert a strong stimulatory influence on muscle ANGPT2 expression while pro-inflammatory cytokines do not. ANGPT2 promotes skeletal myoblast survival and differentiation. These results suggest that muscle-derived ANGPT2 production may play a positive role in skeletal muscle fiber repair.

## Introduction

Through their specific effects on precursor cell survival and adhesion, angiopoietins are recognized not only as angiogenesis regulators but also as important modulators of skeletal muscle function [1]. Angiopoietins are ligands for TIE2 receptors. Angiopoietin-1 (ANGPT1) is the main ligand that is produced, mainly by cardiac skeletal and smooth muscle cells, adventitial cells, and, to a lesser extent, endothelial cells [2]. In comparison, angiopoietin-2 (ANGPT2) is expressed in the endothelium, tumor cells, macrophages and muscle cells [3]–[6]. In endothelial cells, ANGPT2 binds to TIE2 receptors with similar affinity to that of angiopoietin 1 (ANGPT1). However, while ANGPT1 is a strong agonist of TIE2 receptors, the ability of ANGPT2 to activate TIE2 receptors is highly dependent on the cell type and context [2].

Most investigators agree that ANGPT1 and TIE2 receptors play critical roles in early vascular development and angiogenesis. The involvement of ANGPT2 is less clear, particularly in regard to embryonic vascular and lymphatic formation. The observation that transgenic overexpression of ANGPT2 elicits similar phenotypes to those seen in TIE2- and ANPGT1-deficient mice has led to the conclusion that ANGPT2 antagonizes ANGPT1 during vascular development [5]. However, a recent study using ANGPT2-deficient mice has revealed that ANGPT2 plays a crucial role in lymphatic vascular development, as indicated by post-natal lethality, the development of chylous ascites and the appearance of lymphatic vascular defects [7].

In the vascular system, ANGPT2 expressions are significantly induced by pro-inflammatory mediators. Indeed, exposure to TNFα, thrombin or angiotensin II upregulates ANGPT2 expression in cultured endothelial cells [8], [9]. It has also been documented that plasma ANGPT2 levels are elevated in patients with severe sepsis and in children with septic shock, and injection of ANGPT2 protein *in vivo* elicits significant increases in edema formation in the mouse paw [10]–[12]. These findings suggest a strong link between inflammatory mediators, the development of oxidative stress (important contributor to tissue injury in sepsis) and ANGPT2 expression in the vascular system.

In contrast, however, to what is known about vascular ANGPT2 expression, little is known about the presence and expression of ANGPT2 in non-vascular cells, including in skeletal muscles. For example, it is not clear which factors regulate ANGPT2 expression in these muscles. The first objective of this study, therefore, is to document ANPGT2 expression in skeletal muscle cells and to evaluate the roles of pro-inflammatory cytokines and reactive oxygen species (ROS) on skeletal muscle-derived ANPGT2 production. Our main hypotheses are that skeletal muscle cells constitutively express ANGPT2 and that muscle-derived ANGPT2 production is significantly induced by pro-inflammatory cytokines and ROS. This hypothesis is based on the strong relationships between induction of ANGPT2 production and elevated pro-inflammatory cytokine levels in the vascular system and on the observation that ROS trigger the release of angiogenesis factors in skeletal muscle cells [10]–[13].

Skeletal muscle precursor cells are muscle-specific progenitor cells that play a critical role in skeletal muscle regeneration in response to injury. They proliferate, migrate, fuse to each other then differentiate to form myofibers, leading to complete regeneration of muscle fibers. Many angiogenic growth factors, such as VEGF, FGFs, and insulin-like growth factor-1 (IGF1), act in an autocrine fashion to promote precursor cell myogenesis by enhancing proliferation, migration, differentiation and survival of these cells [14]–[16]. Although signaling and vascular effects of endothelial cell angiopoietins are well characterized, there is as yet a paucity of information regarding the influence of angiopoietins on the myogenesis program of skeletal muscle precursor cells. The second objective of this study, therefore, is to evaluate functional effects of ANGPT2 on skeletal muscle precursor cell myogenesis, including proliferation, migration, differentiation and survival, and the mechanisms through which ANGPT2 influence these processes. On the basis of preliminary experiments, we hypothesize that ANGPT2 plays positive roles in promoting both differentiation and survival of muscle cells and that these effects are mediated through selective activation of specific cellular signaling pathways that include protein kinase B (AKT) and the ERK1/2 members of the mitogen-activated protein kinases (MAPKs).

## Materials and Methods

### ANGPT2 Expression in skeletal and endothelial cells

While ANGPT2 is known to be produced by endothelial cells, production of this factor by primary skeletal precursor cells is less clear. Hence, we measured the levels of ANGPT2 production in both, human and murine primary skeletal precursor cells. Primary human muscle precursor cells (human myoblasts) immortalized by expression of the E6E7 early region from human papillomavirus type 16, were generously provided by Dr. E. Shoubridge (McGill University, Montréal, QC), and cultured in SkBM culture medium (SkBM® Bullet Kit, Cambrex, East Rutherford, NJ) supplemented with 15% inactivated fetal bovine serum (FBS)[17]. Cells were collected and total RNA was extracted using a GenElute™ Mammalian Total RNA Miniprep Kit (Sigma-Aldrich, Oakville, ON). Quantification and purity of total RNA was assessed by A260/A280 absorption. The levels of ANGPT2 mRNA and 18S (control gene) were detected using TaqMan real-time PCR assays (Applied Biosystems Inc.). RNA (2 µg) was reverse- transcribed using Superscript II® Reverse Transcriptase Kits and random primers (Invitrogen Inc.). Reactions were incubated at 42°C for 50 min and at 90°C for 5 min. Real-time PCR was performed using a Prism 7000 Sequence Detection System (Applied Biosystems, Foster City, CA). To quantify expressions of ANGPT2 and 18S (endogenous control), TaqMan® Gene Expression Assays (Applied Biosystem Inc.) specific to these genes were used. The thermal profile was as follows: 95°C for 10 min and 40 cycles of 95°C for 15 sec, 57°C for 30 sec, and 72°C for 33 sec. All real-time PCR experiments were performed in triplicate. A melt analysis for each PCR experiment was performed to assess primer-dimer formation or contamination. To determine the absolute copy numbers of ANGPT2 and 18S mRNA transcripts, standard curves that related the cycle threshold (CT) values of these genes to the copy numbers were established, as described in our recent study [3]. Copy numbers of ANGPT2 were then normalized per copies of 18S. In addition, relative quantification of ANGPT2 mRNA levels compared to those of control condition was determined using the threshold cycle (ΔΔCT) method.

To evaluate the changes in ANGPT2 mRNA in human myoblasts during differentiation to myotubes, human skeletal myoblasts were induced to differentiate into myotubes by growing confluent myoblasts (90% confluent) in Dulbecco's modified Eagle's medium (DMEM) (Invitrogen Inc., Carlsbad, CA) supplemented with 2% inactivated horse serum (HS) for 7 days. Cells were collected at days 0 (myoblasts), 1, 3, 5 and 7 and total RNA was extracted as described above. ANGPT2 mRNA and 18S levels during differentiation were detected using real-time PCR as described above.

All animal experiments were approved by McGill University Animal Care Committee (protocol# 3870). To isolate primary murine skeletal precursor cells, tibialis anterior muscle strips were extracted from 6-week-old C57/BL6 mice, digested with collagenase (0.2% at 37°C for 60 min) and then triturated to break muscle tissues into single fibers. Individual fibers were washed in (DMEM) and phosphate-buffered saline, transferred into Matrigel®-coated (1 mg/ml in DMEM) 6-well plates and maintained in DMEM supplemented with 1% penicillin/streptomycin and 0.2% amphotericin B, 10% HS and 0.5% chick embryo extract (MP Biomedicals, Aurora, OH) for 4 days, which allowed myoblasts to attach to the substratum. Cells were then collected and total RNA was extracted as described. ANGPT2 mRNA levels were detected using real-time PCR and murine Taqman® ANGPT2 assays (Applied Biosystems). ANGPT2 mRNA expression in these cells was expressed as copies normalized for 18S copies.

### Regulation of ANGPT2 expression in human myoblasts by cytokines and H_2_O_2_

To assess whether muscle-derived ANGPT2 production is under the control of inflammatory cytokines and oxidagive stress, we measured the effects of TNFα, IL1β, IL6 and hydrogen peroxide (H_2_O_2_, mediator of oxidative stress) on ANGPT-2 expression in muscle cells. Human skeletal myoblasts were plated into 12-well plates (10^5^ cells per well) and maintained for 24 h in SkBM culture medium containing 5% FBS. Cells were then stimulated with recombinant human IL-1β (20 ng/ml), TNFα (40 ng/ml), IL6 (50 ng/ml), mixture of the three cytokines, H_2_O_2_ (0.5 mM), or a control solution of phosphate-buffered saline (PBS). Cells were collected 24 h later and prepared for total RNA extraction and ANGPT2 mRNA expression using real-time PCR (see above). Relative quantification of ANGPT2 mRNA levels in cells treated with cytokines or H_2_O_2_ compared to control cells was determined using the threshold cycle method. In additional experiments, human myoblasts were stimulated for 24 h with TNFα (40 ng/ml) or H_2_O_2_ (0.5 mM) or PBS. Media and cell lysates were collected ANGPT2 protein levels in the media were measured with ELISA (Angiopoietin-2 Quantikine ELISA, R& D Systems Inc.) and normalized for cell lysate protein levels.

### Regulation of ANGPT2 expression in human umbilical vein endothelial cells (HUVECs)

To compare the influence of ANGPT2 on survival of skeletal precursor cells to that elicited in endothelial cells, we studied HUVECs which were cultured in endothelial basal medium (MCDB131) supplemented with 20% fetal bovine serum (FBS), endothelial cell growth supplement, 2 mM glutamine, heparin, penicillin (100 units/ml), streptomycin (100 µg/ml) and amphotericin B (0.25 µg/ml). HUVECs were plated into 12-well plates (10^5^ cells per well) and maintained for 24 h in endothelial cell basal medium supplemented with 2% FBS containing PBS (control), TNFα (40 ng/ml) or H_2_O_2_ (0.5 mM). Cells and media were then collected and ANGPT2 protein levels in the media were measured with ELISA as described above.

### Regulation of myoblast myogenesis by ANGPT2

#### Myoblast proliferation

The effects of ANGPT2 on myoblast cell proliferation were measured by seeding human myoblasts (1×10^5^ cells) into 6-well culture plates for 24 h in SkBM culture medium containing 15% FBS. Culture medium was then replaced with SkBM culture medium containing 5% FBS and either PBS or 150, 300 and 600 ng/ml of recombinant human ANGPT2. These concentrations have been extensively employed to evaluate the biological responses to ANGPT2 in cultured cells. Culture medium containing PBS or ANGPT2 protein was replenished every 24 h. After 72 h of exposure, cells were then exposed to 0.5% trypsin-EDTA, stained with trypan blue and viable cells were counted by hematocytometer.

#### Myoblast migration

Regulation of skeletal myoblast migration by ANGPT2 was assessed using wound healing assay. Human myoblasts (1×10^5^ cells) were seeded into 6-well culture plates and maintained for 24 h in SkBM culture medium containing 15% FBS. Cells were then carefully wounded using a 200-µl pipette tip, as previously described [18]. Cellular debris was removed by washing with phosphate-buffered saline (PBS). After wounding, culture medium was replaced with SkBM containing 5% FBS and either PBS (control) or 600 ng/ml recombinant human ANGPT2. Wounds were photographed immediately after wounding (time = 0) and 12 and 18 h later using an Olympus inverted microscope (Model X70) equipped with phase-contact lenses. Migration was evaluated by measuring the reduction in the diameter of the wound after migration of the cells into the cell-free zone [3726}. For each condition, three wells of a given 6-well plate were used, and the procedure was performed in triplicate.

#### Intracellular signaling by ANGPT2 in satellite cells

Several reports have confirmed that the PI-3 kinase/AKT and the Erk1/2 pathways are important in promoting many of the biological effects of angiopoietins in endothelial cells [19], [20]. To evaluate whether these two pathways are activated by ANGPT2 in skeletal muscle precursors, human skeletal myoblasts were maintained in SkBM culture medium containing 5%FBS for 12 h. Cells were then stimulated with PBS (control) or recombinant human ANGPT2 (300 ng/ml) for 5, 15, 30 and 60 min. The medium was then removed and adherent cells were washed twice with PBS and lysed in basic lysis buffer (see below). In addition, human myoblasts were stimulated for 15 min with PBS or 50, 150, 300 or 600 ng/ml of ANGPT2. Cells were lysed in basic lysis buffer containing HEPES (50 mM), NaCl (150 mM), NaF (100 mM), EDTA (5 mM) and protease inhibitors (aprotinin 5 mg/ml, leupeptin 2 mg/ml and PMSF 100 mM). Cell debris and nuclei were separed by centrifugation at 14,000 g for 5 min at 4°C. Supernatants were then boiled for 5 min then loaded onto tris-glycine SDS-PAGE. Proteins were electrophoretically transferred onto polyvinylidene difluoride membranes, blocked for 1 h with 5% nonfat dry milk, and incubated overnight at 4°C with primary antibodies to phospho-AKT (Thr^308^), total AKT, phospho-Erk1/2 (Thr^202^/Tyr^204^) and total Erk1/2 (Cell Signaling Inc.). Proteins were detected with horseradish peroxidase–conjugated secondary antibodies and ECL reagents (Chemicon). Loading of equal amounts of protein was confirmed by stripping the membranes and re-probing with anti-tubulin antibody (Sigma-Aldrich). Blots were scanned with an imaging densitometer, and optical densities of protein bands were quantified with ImagePro software (Media Cybernetics, Carlsbad, CA). Predetermined molecular weight standards were used as markers. Protein concentrations were measured by the Bradford method, with BSA as a standard.

#### Myoblast survival and apoptosis

To evaluate the influence of ANGPT2 on skeletal muscle precursor cell survival, human skeletal myoblasts (25×10^3^ cells) were seeded into 96-well plates and maintained for 12 h in SkBM culture medium containing 15%FBS. Culture medium was then replaced with SkBM culture medium (0%FBS) containing either PBS or 300 ng/ml recombinant human ANGPT2. Cell cytotoxicity and caspase3/7 activity were measured 36 h later using CytoTox-Fluor™ Cytotoxicity Assay and Caspase-Glo™ 3/7 Assay, respectively, according to the manfacturer's instructions (Promega Inc. Madison, WI). The involvement of the PI-3 kinase/AKT and Erk1/2 pathways in the regulation of muscle cell survival and caspase 3/7 activity, we incubated human skeletal myoblasts in SkBM culture medium (0%FBS) containing either PBS (control condition) or 300 ng/ml of recombinant human ANGPT2 in the presence and absence of 2 µM of PD184352 (Erk1/2 inhibitor), 50 nM of wortmannin (PI-3 kinase inhibitor) and 1 µM of API-2 (Tricirbine, AKT inhibitor). Cell death (cytotoxicity) and caspase3/7 activity were measured 36 h later using CytoTox-Fluor™ Cytotoxicity Assay and Caspase-Glo™ 3/7 Assay, respectively.

#### Myoblast differentiation

We evaluated the influence of ANGPT2 on human myoblast differentiation by over-expressing ANGPT2 in human skeletal myoblasts using adenoviruses. The choice of using adenoviruses to deliver ANGPT2 was aimed at achieving sustained endogenous elevation of ANGPT2 production over several days required for full differentiation of skeletal myoblasts into myotubes. Recombinant adenoviruses expressing ANGPT2 (Ad-ANGPT2) were constructed using the Adeno-Quest™ system (Quantum Biotechnology Inc., Montreal, Canada). Full-length cDNA encoding human ANGPT2 was cloned into shuttle vector pQBI-AdCMV5GFP [21]. Sub-confluent human myoblasts were maintained for 6 h in SkBM culture medium containing 250 MOI (multiplicity of infection) of Ad-ANGPT2 or Ad-GFP (control cells). Cells were then washed and maintained for 48 h in SkBM culture medium containing 15% FBS to reach full confluence. After this recovery period, the cells were collected (day 0, myoblast phase) for RNA extraction and also differentiated into myotubesby replacing the medium with Dulbecco's modified Eagle's medium (DMEM) (Invitrogen Inc., Carlsbad, CA) supplemented with 2% inactivated horse serum (HS). Differentiating cells were collected at 1, 3, 5 and 7 days of incubation with the differentiation medium. Total RNA was extracted as described above. Total RNA (2 µg) was then reverse transcribed using Superscript II® Reverse Transcriptase Kits and random primers (Invitrogen). Reactions were incubated at 42°C for 50 min and at 90°C for 5 min. Expression of myogenic transcription factors MyoD and Myogenin as well as muscle-specific Myosin Heavy Chain and Creatine Kinase mRNA levels as well as that of 18S levels during differentiation were detected using real-time PCR as described above. Primers designed to amplify these transcripts are listed in Supporting Information S1. Real-time PCR was performed using a Prism 7000 Sequence Detection System (Applied Biosystems, Foster City, CA). To determine the absolute copy numbers of mRNA transcripts of a specific gene and 18S mRNA transcripts, standard curves that related the cycle threshold (CT) values of these genes to the copy numbers were established, as described in our recent study [3]. Copy numbers of these genes were then calculated as described above and normalized per copies of 18S.

### Mechanisms of ANGPT2 action in skeletal myoblasts

To investigate the mechanisms through which ANGPT2 regulates skeletal myoblast survival and differentiation, we over-expressed human ANGPT2 in human skeletal myoblasts using adenoviruses (Ad-ANGPT2) and compared mRNA expression profiles of these cells to those infected with control adenoviruses (Ad-GFP). Sub-confluent human myoblasts were maintained for 6 h in SkBM culture medium containing 250 MOI (multiplicity of infection) of Ad-ANGPT2 (n = 6) or Ad-GFP (n = 6). Cells were then washed and maintained for 48 h in SkBM culture medium containing 15% FBS. After this recovery period, the culture medium was changed to SkBM culture medium containing 5% FBS. Cells were collected 12 h later and total RNA was extracted as described above.

#### Illumina microarrays

Total RNA (100 ng) was amplified using the Illumina RNA Amplification kit (Illumina Inc., San Diego CA) and labelled by incorporation of biotin-16-UTP. Samples were hybridized to Sentrix Genome-Wide Expression BeadArray™ (Illumina Inc.). These arrays use beads containing 50-mer gene-specific probes (a total of 46,000 probes per array). Arrays were scanned with an Illumina BeadArray Reader (Montreal Genome Centre, Montreal, Canada) and data processing and normalization were performed using Illumina Bead-Studio software. Signal values were normalized by global mean and log transformed using GeneSifter software (VizX Labs, Seattle, WA, USA). Pairwise comparisons and ANOVA were subsequently performed using FlexArray 1.1.3 sofware package (Genome Quebec, Montreal, Quebec, Canada) and a difference of at least two-fold with a P-value of less than 0.05 was considered as a statistically significant change in gene expression. All data is MIAME compliant and the raw data has been deposited in a MIAME compliant database (GEO). To investigate gene networks regulated by ANGPT2 in cultured human skeletal myoblasts, we analyzed the microarray results using Ingenuity Pathway Analysis which is a web-based bioinformatics tool (IPA, http://www.ingenuity.com) based on >1.7 Million published articles. IPA is a knowledge database generated from the peer-reviewed scientific publications that enables discovery of biological networks in gene expression data, determining the functions most significant to those networks. Gene name identifiers or Ingenuity probe set ID's were uploaded into IPA and queried against all other genes stored in the IPA knowledge database. Each Ingenuity probe set ID was mapped to its corresponding gene identifier in the IPA knowledge database. Probe sets representing genes having direct interactions with genes in the IPA knowledge database are called “focus” genes, which were then used as a starting point for generating functional networks. Each generated network is assigned a score according to the number of differentially regulated focus genes in our array dataset. These scores are derived from negative logarithm of the P indicative of the likelihood that focus genes found together in a network due to random chance. Scores of 14 or higher have 99.9% confidence level of significance. In reporting our findings we list networks with a substantially higher confidence limit and thus represent strong evidence for a given biological pathway being regulated by ANGPT2 in human skeletal myoblasts. It should be noted however that while the database extends the interpretation beyond mRNA transcript levels (as network genes don't have to be differentially expressed at the mRNA level) the database is finite and reflects current knowledge.

To confirm the results obtained with the microarrays, we performed real-time PCR to evaluate the expression of four genes which were found to be upregulated (TEL2 and LEP) and downregulated (CSF3, ANGPTL4 and CTGF) in cells infected with Ad-ANGPT2 compared to cells infected with Ad-GFP. Real-time PCR was performed as described above using primers listed in Supporting Information S1. Relative quantification of TEL2, LEP, CSF3, ANGPTL4 and CTGF mRNA levels in cells infected with Ad-ANGPT2 compared to cells infected with Ad-GFP was determined using the threshold cycle (ΔΔCT) method. In addition, we verified enhanced leptin production in muscle cells by directly measuring leptin levels in the culture medium. Sub-confluent human myoblasts were maintained for 6 h in SkBM culture medium containing 250 MOI (multiplicity of infection) of Ad-ANGPT2 (n = 6) or Ad-GFP (n = 6). Cells were then washed and maintained for 72 h in SkBM culture medium containing 5% FBS. The levels of secreted leptin in the culture medium were measured with a commercial leptin ELISA kit from R&D Systems.

#### Pathways regulated by ANGPT2

Data were analyzed using the IPA (Ingenuity® Systems, www.ingenuity.com). A data set containing gene identifiers and corresponding expression values was uploaded into the application. Each gene identifier was mapped to its corresponding gene object. A fold-change cutoff of 2 for both up- and down-regulation and a p-value cutoff of 0.05 were set to identify the genes to be analyzed. These genes, called focus molecules, were overlaid onto a global molecular network developed from information in the IPA. Networks of these focus molecules were then algorithmically generated based on their connectivity. The functional analysis of a network identified the biological functions and/or diseases that were most significant to the genes in the network. Genes and gene products are represented as nodes, and the biological relationship between two nodes is represented as an edge (line). All edges are supported by at least one reference from the literature, textbook or canonical information stored in the IPA. Human, mouse and rat orthologs of a gene are stored as separate objects in the IPKB, but are represented as a single node in the network. The node color indicates the degree of up- (red) or down- (green) regulation. Nodes are displayed using various shapes that represent the functional class of the gene product. It should be emphasized that one round of analyses were performed with the Ingenuity Pathway Analysis system considering both up- and downregulated genes in cells infected with Ad-ANGPT2 compared with cells infected with Ad-GFP.

### Statistical analyses

Results are expressed as means ±SE in all figures. Differences in terms of cell proliferation, migration, cell death, caspase 3/7 activity, and muscle-specific gene mRNA levels during the differentiation protocols were detected with Analysis of Variance with a Bonferroni-type adjustment. Values of P<5% were considered significant.

## Results

### ANGPT2 expression in skeletal and endothelial cells

**Figure 1:**
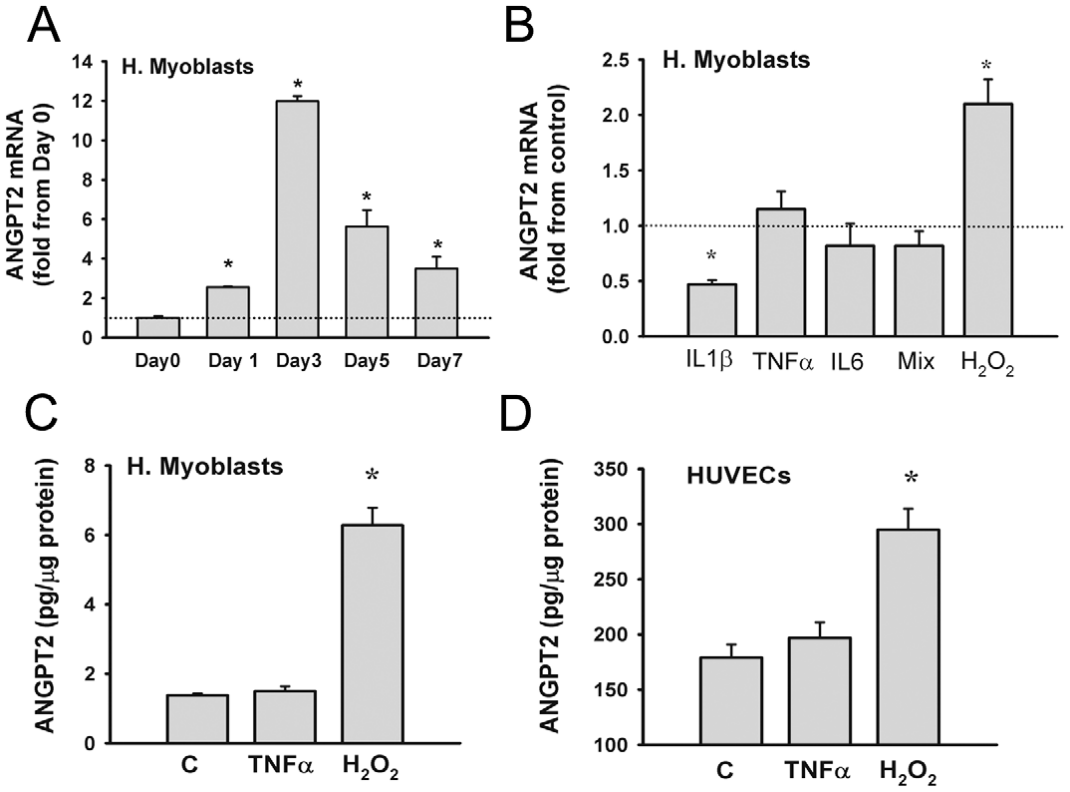
Regulation of ANGPT2 expression in myoblasts. **A:** mRNA expression of ANGPT2 in human skeletal myoblasts (day 0) and after 1, 3, 5 and 7 days of differentiation into myotubes. Values are means ± SE. *P<0.05 compared with day 0. **B:** ANGPT2 mRNA expression in human skeletal myoblasts exposed for 24 h to IL1β, TNFα, IL6, a combination of the three, or H_2_O_2_. N = 6. *P<0.05 compared with control. **C**: ANGPT2 protein levels in the media of human skeletal myoblasts exposed for 24 h to BSA (control, C), TNFα or H_2_O_2_. N = 6. *P<0.05 compared with control. **D**: ANGPT2 protein levels in the media of human umbilical vein endothelial cells (HUVECs) exposed for 24 h to PBS (control, C), TNFα or H_2_O_2_. N = 6. *P<0.05 compared with control.

In primary human myoblasts ANGPT2 mRNA levels are detectable at an abundance of 0.24±0.04 copies/10^3^ copies of 18S. Those values are similar to those detected in murine skeletal myoblasts (0.25±0.03 copies/10^3^ copies of 18S). Expression of ANGPT2 mRNA in human myoblasts is significantly induced when they differentiate into myotubes, indicating that mature muscle fibers produce higher levels of ANGPT2 than their precursors (Figure 1A). Figure 1B indicates that ANGPT2 mRNA levels in human myoblasts were significantly reduced by IL1β, significantly augmented by H_2_O_2_ whereas TNFα and IL6 had no effects. Lack of TNFα effect on ANGPT2 production and upregulation of this production by H_2_O_2_ were verified by measuring ANGPT2 protein levels in the media of human myoblasts and human umbilical vein endothelial cells (Figure 1C and D).

### Regulation of myoblast myogenesis by ANGPT2

**Figure 2:**
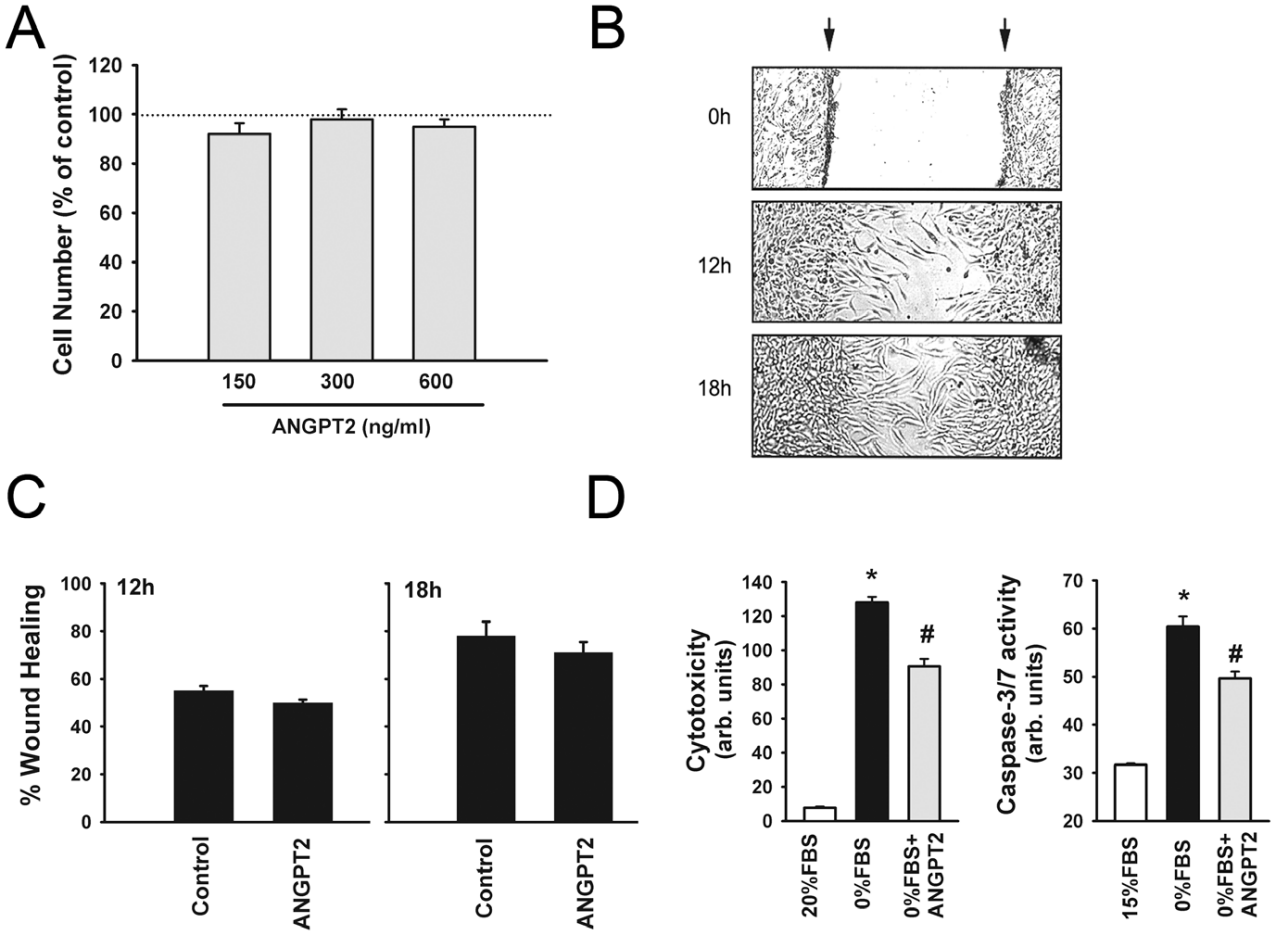
Regulation of myoblast proliferation, migration and survival by ANGPT2. **A:** Cell number of human skeletal myoblasts exposed to PBS (control) or ANGPT2. Cells maintained for 72 in culture medium. N = 6. **B:** Representative Photographs of wound healing assays in human skeletal myoblasts. Arrows indicate margins of wounds. **C:** Wound healing intensity in myoblasts exposed to PBS (control) or ANGPT2. N = 6 per group. **D:** Cytotoxicity and caspase 3/7 activity in human skeletal myoblasts maintained for 36 h in media containing 15%FBS, 0%FBS or 0%FBS + ANGPT2. N = 8 per group. *P<0.05 compared with 15% FBS, ^#^P<0.05 compared with 0% FBS.

**Figure 3:**
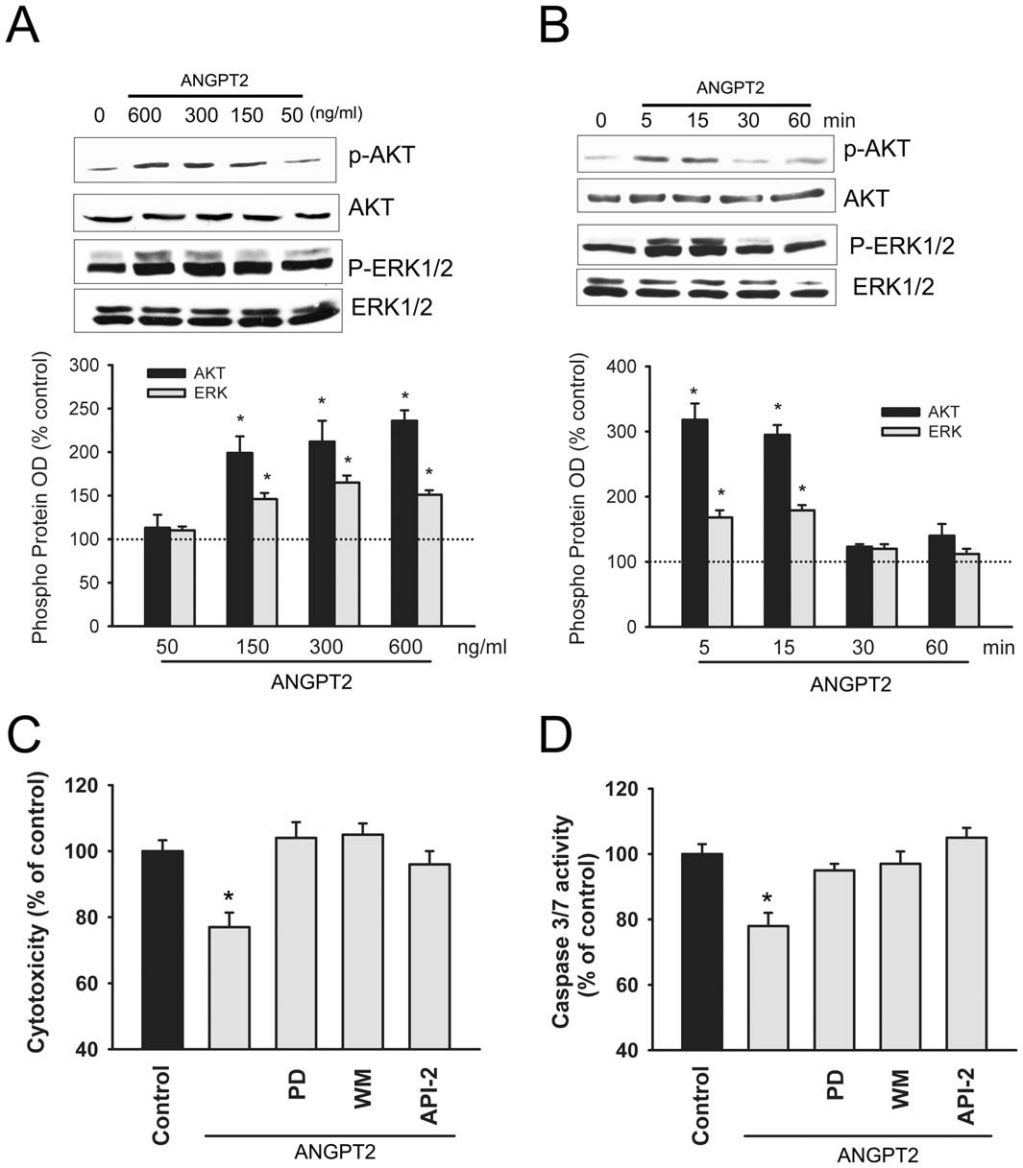
ANGPT2 signaling in skeletal myoblasts. **A:** Representative immunoblots and optical densities of total and phosphorylated AKT and ERK1/2 proteins in human skeletal myoblasts exposed for 15 min to increasing concentrations of ANGPT2. N = 4 per group. **B:** Representative immunoblots and optical densities of total and phosphorylated AKT and ERK1/2 proteins in human skeletal myoblasts exposed to 600 ng/ml ANGPT2 for increasing durations. N = 4 per group. **C–D:** Cytotoxicity and caspase 3/7 activity in human skeletal myoblasts maintained for 36 h in media containing 0%FBS (control) or 0%FBS + ANGPT2 in the presence of PD184352, (PD, ERK1/2 inhibitor), wortmannin, (WM, PI3 kinase inhibitor) and API2 (AKT inhibitor). *P<0.05 compared with control.

To evaluate the functional significance of increased ANGPT2 expression on muscle fiber regeneration, we measured the effects of exogenous ANGPT2 on proliferation, migration, differentiation and survival of human skeletal myoblasts. ANGPT2 has no effect on myoblast proliferation and migration (Figure 2A–C). However, ANGPT2 significantly reduces cytotoxicity and capase-3/7 activity in skeletal myoblasts exposed to complete serum withdrawal, indicating that ANGPT2 promotes myoblast survival (Figure 2D). To investigate the signaling pathways through which ANGPT2 promotes myoblast survival, we evaluated the effects of ANGPT2 on activation of two important pro-survival pathways (PI3 kinase/AKT and ERK1/2). ANGPT2 elicited transient and dose-dependent increases in AKT phosphorylation on Thre^308^ (site of PI3 kinase mediated phosphorylation) and ERK1/2 phosphorylation on Thre^202^/Tyr^204^ (site of ERK1/2 mediated phosphorylation (Figure 3A and B). The importance of the PI3 kinase/AKT and ERK1/2 pathways in the pro-survival effects of ANGPT2 was assessed using selective pharmacological inhibitors of these pathways. The inhibitory effects of ANGPT2 on serum deprivation-induced cytotoxicity and caspase 3/7 activity are completely eliminated in the presence of selective inhibitors of PI3 kinase, AKT and Erk1/2 (Figure 3C and D). It should be noted that these inhibitors by themselves had no significant effects on cytotoxicity and caspase 3/7 activity. Our results suggest that ANGPT2 promotes myoblast survival and inhibits apoptosis in these cells through the PI3 kinase/AKT and Erk1/2 pathways.

**Figure 4:**
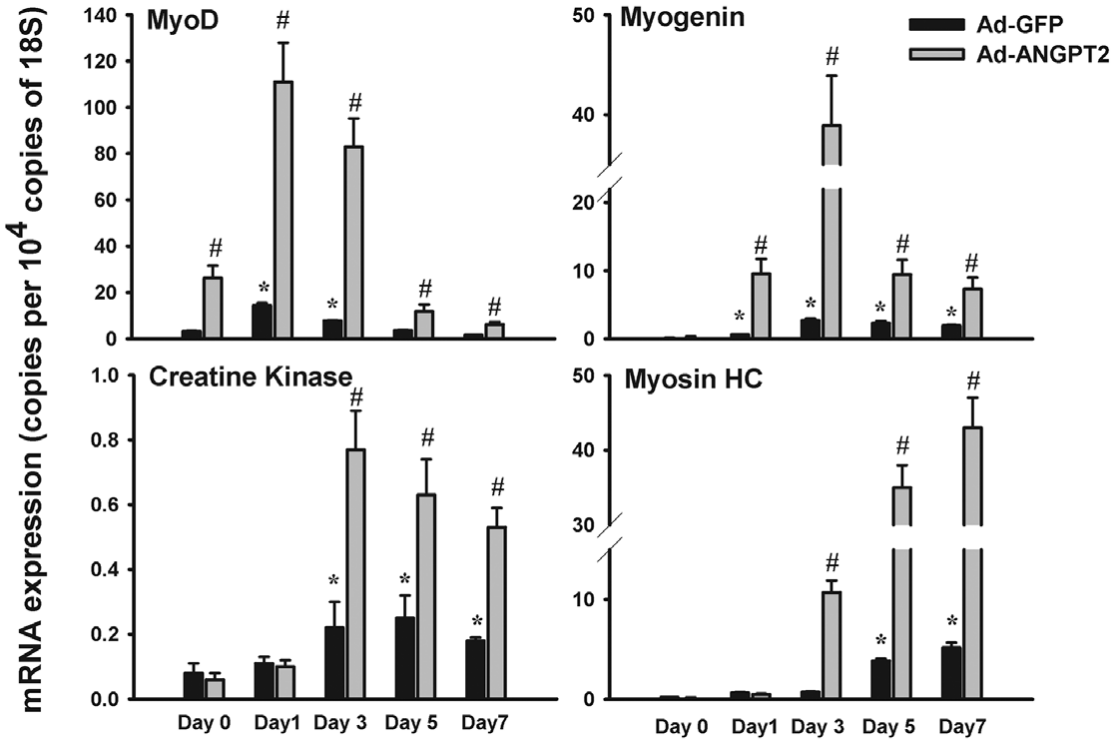
Regulation of myoblast differentiation by ANGPT2. mRNA expressions of MyoD, Myogenin, Creatine Kinase and Myosin Heavy Chain in human skeletal myoblasts infected with Ad-GFP (control) or Ad-ANGPT2 viruses. Cells were collected at the myoblast phase (day 0) and after 1, 3, 5 and 7 days of differentiation into myotubes. N = 6 per group. *P<0.05 compared with day 0 (myoblast phase). # P<0.05 compared with cells infected with Ad-GFP.

Figure 4 shows the influence of ANGPT2 on differentiation of human skeletal myoblasts. Significant inductions of MyoD and Myogenin occur in control adenovirus (Ad-GFP) transfected cells incubated for 1 day in differentiation medium; Creatine Kinase and Myosin Heavy Chain expressions are significantly induced after 3 and 5 days of incubation, respectively (Figure 4). In myoblasts transfected with Ad-ANGPT2, even greater levels of differentiation take place, as indicated by the earlier and significantly higher expression levels of MyoD, Myogenin, Creatine Kinase and Myosin Heavy Chain genes, as compared to cells infected with Ad-GFP (Figure 4).

### Mechanisms of ANGPT2 action in skeletal myoblasts

The transcriptomes of human skeletal myoblasts infected with adenoviruses expressing GFP (control) and ANGPT2 were compared using Illumina microarrays which revealed that expressions of 45 genes were significantly upregulated by ANGPT2 in skeletal myoblasts (Table 1 and Supporting Information S1). The largest relative increase was detected in NRP1 (receptor for VEGF family of proteins) and TELO2 (regulator of cell cycle). ANGPT2 also induced the expression of several other genes involved in cell signaling, including LEP (leptin) (Table 1 and Supporting Information S1).

**Table 1:** Summary of genes whose expression is significantly altered in Ad-ANGPT2-infected myoblasts as compared with cells infected with Ad-GFP viruses.

Genes induced by ANGPT2 in skeletal myoblasts				
Accession	Symbol	Definition	Ad-ANGPT2/	Function
			Ad-GFP	
NM001024629	NRP1	Neuropilin 1 transcript variant 3	12.9	Angiogenesis
NM016111	TELO2	Telomere maintenance 2 homology	12.0	Cell cycle
NM201414	APP	Amyloid beta A4 precursor	8.9	Apoptosis
NM033397	ITPRIP	Inositol triphosphate interacting protein	3.2	Signaling
NM032340	C6orf125	Chromosome 6 open reading frame 125	3.1	Carbohydrase transport
NM000230	LEP	Leptin	3.1	Signaling
NM182480	COQ6	Coenzyme Q6 homolog	2.9	Electron transport
NM181877	ZSCAN2	Zinc finger and SCAN domain containing 2	2.8	Transcription
NM032645	C10orf33	Chromosome 10 open reading frame 33	2.7	Electron transport
NM032645	RAPSN	Receptor-associated protein of synapse	2.6	Proteolysis
NM016401	C11orf73	Chromosome 11 open reading frame 73	2.6	Unknown
NM001011539	LOC44104	Hypothetical LOC441046	2.5	Metabolism
NM199295	APITD1	Apoptosis-induced TAF9-like domain 1	2.5	Transcription
**Genes inhibited by ANGPT2 in skeletal myoblasts**				
NM002155	HSPA6	Heat shock 70 kDA protein 6	0.03	Protein folding
NM178539	FAM19A2	Family with sequence similarity 19	0.05	Signaling
NM001012977	PAP1M	Polyadenylate-binding protein 1	0.05	RNA stability
NM005345	HSPA1A	Heat shock 70 kDA protein 1A	0.16	Protein folding
NM139016	C20orf198	Chromosome 20 open reading frame 198	0.20	Unknown
NM005346	HSPA1B	Heat Shock 70 kDa protein	0.21	Protein folding
NM001002796	MCTP1	Multiple C2 domains, transmembrane 1	0.21	Ca++-mediated signaling
NM005252	FOS	FBJ murine osteosarcome oncogene homolog	0.22	Transcription
NM032461	SPANXB1	Spanx family, member B1	0.26	Transcription
NM153292	NOS2A	Nitric oxide synthase 2A	0.26	Signaling
NM030926	ITM2C	Integral membrane protein 2C	0.26	Unknown
NM013453	SPANXA1	Spanx family member A1	0.27	Transcription
NM000488	SERPINC1	Serine peptidase inhibtor, clade C	0.27	Apoptosis
NM172220	CSF3	Colony stimulating factor 3	0.28	Immune responses
NM018602	DNAJA4	DNAJ (Hsp40) homolog	0.28	Protein folding
NM022661	SPANXC	Spanx family member C	0.28	Transcription
NM001005611	EDA	Ectodysplasin A	0.29	Immune responses

Table 1 and Supporting Information S1 list 95 genes whose expression is significantly downregulated by ANGPT2. Heat shock 70 kDa proteins, SPANX transcription factors, inducible nitric oxide synthase (iNOS), FOS and FOBSB subunits of activating protein 1 (AP1) transcription factor and colony stimulating factor 3 are the most strongly inhibited genes. ANGPT2 also inhibited the expression of IL6, the chemokine CLL26, angiopoietin-like 4, forkhead transcription factor FOXF2, connective tissue growth factor (CTGF) and insulin receptor substrate 4 (Table 1 and Supporting Information S1).

**Figure 5:**
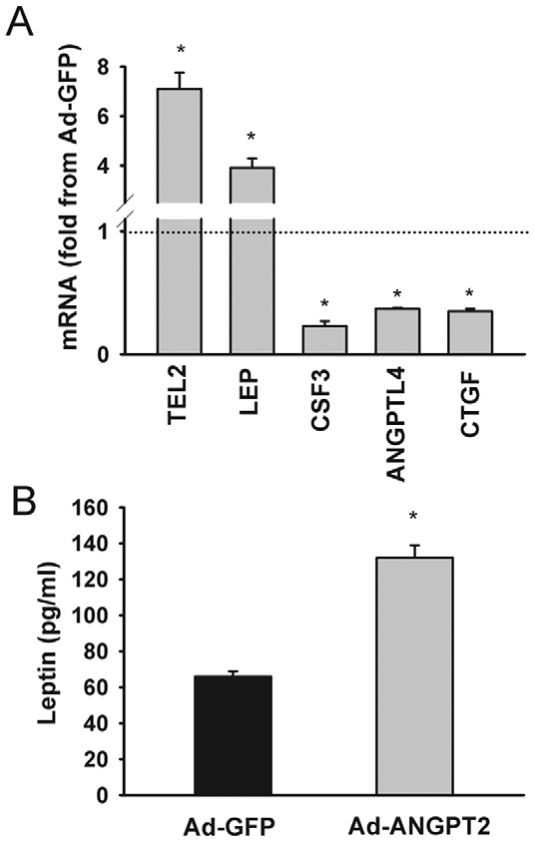
Gene regulation by ANGPT2 in skeletal myoblasts. **A:** Expressions of TELO2, LEP, CSF3, ANGPTL4 and CTGF in human skeletal myoblasts infected with Ad-ANGPT2 viruses. Values are expressed as fold changes from those measured in cells infected with Ad-GFP (control). **B:** Expression of leptin in human skeletal myoblasts infected with Ad-GFP (control) and Ad-ANGPT2 viruses. *P<0.05 compared with Ad-GFP.

**Figure 6:**
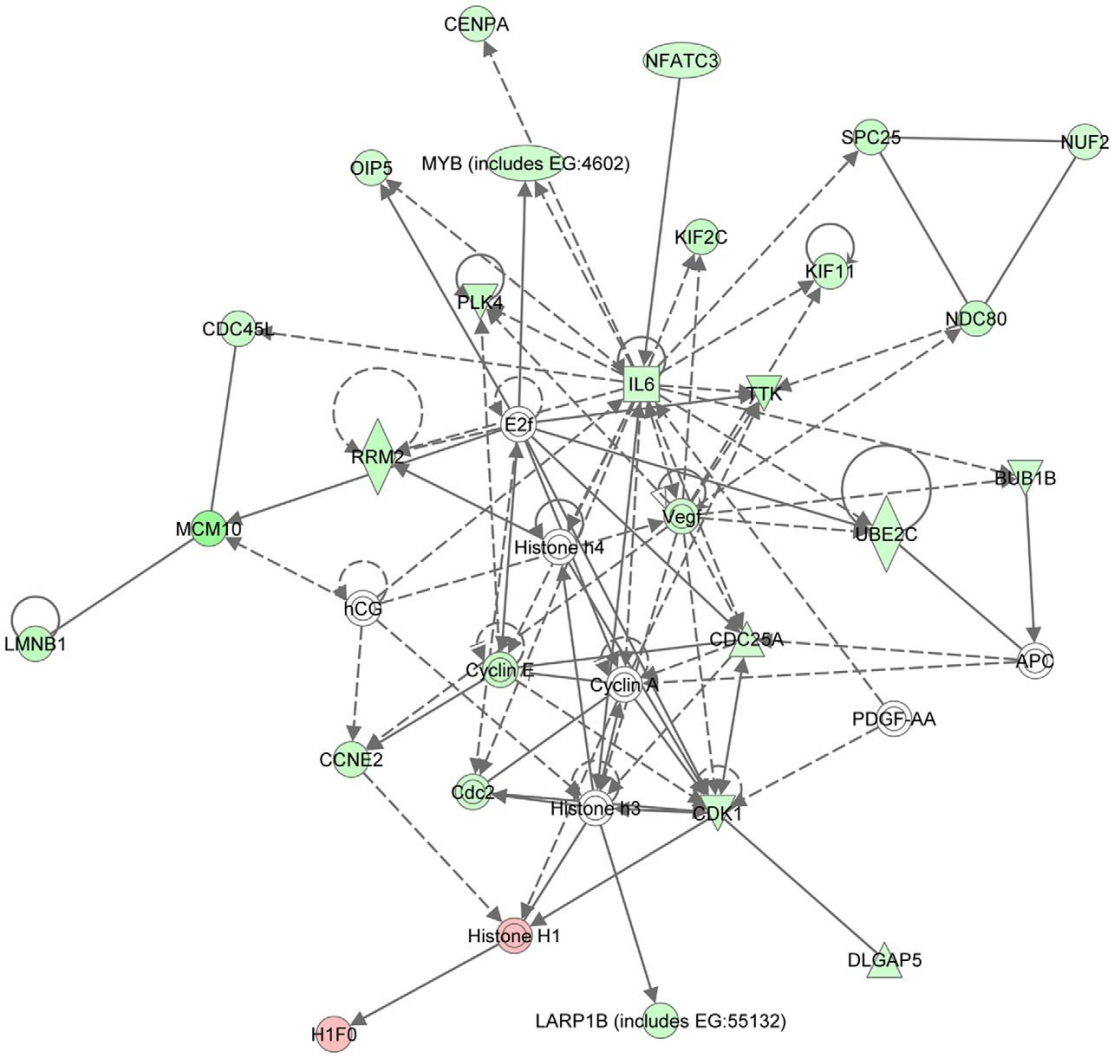
Network analysis of ANGPT2-induced changes in myoblast transcriptome. The top network of regulated genes in human myoblasts infected with Ad-ANGPT2 viruses vs. cells infected with Ad-GFP viruses (control condition). This figure was created with the Ingenuity Pathway Analysis system. Upregulated genes are shown in red while downregulated genes are shown in green. Color intensity indicates the degree of change in gene expression. White nodes are genes whose expression was not significantly changed in cells infected with Ad-ANGPT2 viruses vs. Ad-GFP viruses. Arrows with plain lines indicate direction interactions, and arrows with interrupted lines represent indirect interactions. Top functions associated with this network include cell cycle, cellular assembly and organization, DNA replication, recombination and repair.

Microarray results were verified by detecting gene expressions of two upregulated genes (TELO2 and LEP) and three downregulated genes (CSF3, ANGPTL4 and CTGF) (Figure 5A). Upregulation of LEP (leptin) mRNA expression in cells infected with Ad-ANGPT2 is associated with a significant increase in secreted LEP protein (Figure 5B). Ingenuity Pathway Analysis software was used to analyze the microarray dataset in the context of biological pathways. Supporting Information S1 lists the top networks and top cellular functions that were generated (data supplement). Seven major networks of ANGPT2 regulated genes were detected which contained 24, 18, 14, 11, 10, 10, and 9 focus genes, respectively (Table 2). The top network contains several highly connected nodes, with IL6 being the most highly connected (Figure 6). The second top network contains several highly connected nodes, including two mitogen activated protein kinase pathways (p38 and SAPK/JNK), inducible nitric oxide synthase (NOS2), three immune modulators (IL1, IL12 and IFNα) and NFκB transcription factor (Supporting Information S1). It should be noted that the majority of the genes identified in the first and second top networks were downregulated by ANGPT2.

**Table 2:** Summary of the results of Ingenuity Pathway Analysis of lists of upregulated and downregulated genes in human skeletal myoblasts infected with Ad-ANGPT2 compared with cells infected with Ad-GFP viruses.

Top Networks		
Associated Network Functions	Score	Focus Molecules
Cell cycle, DNA replication, Recombination and Repair	50	24
Tissue Morphology, Lipid Metabolism, Small Molecule Biochemistry	35	18
Cardiovascular System Development and Function, Tissue Development	25	14
Cellular Assembly and Organization	19	11
Cell Morphology, Cellular Development	16	10
Connective Tissue Development and Function	15	10
Cellular Comprise	14	9

Molecular and Cellular Functions	
Name	# Molecules
Cell Cycle	32
Cell Death	42
Cellular Assembly and Organization	25
DNA Replication, Recombination and Repair	29
Cellular Growth and Proliferation	37

## Discussion

The main findings of our study are: 1) ANGPT2 is constitutively expressed in human and murine primary skeletal myoblasts; 2) ANGPT2 expression increases significantly during myoblast differentiation into myotubes; 3) ANGPT2 expression in both, skeletal myoblasts and endothelial cells is induced by H_2_O_2_ but not by TNFα, IL6 and IL1β; 4) Upregulation of ANGPT2 expression in skeletal myoblasts is associated with increased myoblast survival and differentiation. These effects are mediated through activation of the PI3-kinase/AKT and Erk1/2 pathways and through selective and distinct changes in overall cellular gene expression profiles.

### Expressions of ANGPT2 in skeletal muscles

Several investigators have described the detection of ANGPT2 mRNA in skeletal muscle samples obtained from animals or humans [3], [22], [23]. However, the cellular origin of this expression is unclear because various cell types such as skeletal muscle fibers, endothelial, macrophages and nerve fibers present in muscle biopsies can contribute to total muscle ANGPT2 levels. Our group, as well as that of Dallabrida *et al*. [1], [3], have suggested that human and murine skeletal cells do indeed produce ANGPT2. To evaluate this possibility, primary human and murine skeletal myoblasts were isolated, purified and assessed for ANGPT2 production during muscle differentiation. Our results clearly illustrate that ANGPT2 is produced by skeletal myoblasts and that ANGPT2 production increases substantially upon differentiation into myotubes (Figure 1). Another cell type that may contribute to whole skeletal muscle ANGPT2 expression is endothelial cells. Endothelial cells are well-known to produce higher levels of ANGPT2 than do other cells, and are primarily regulated by transcriptional activation of ANGPT2 promoter [5], [24], but also due to the fact that ANGPT2 release can be rapidly achieved from stores inside Weibel-Palade bodies [25].

Previous studies have described linear relationships between plasma ANGPT2 and TNFα concentrations in humans with endotoxemia and sepsis [12], [26]. These findings raise the possibility that muscle ANPGT2 expression is regulated by a direct effect of TNFα on ANGPT2 transcription. Regulation of ANGPT2 expression by TNFα has been studied in endothelial cells with contradictory results. An initial report by Kim *et al*. [9] described significant but transient upregulation of ANGPT2 expression in HUVECs exposed to TNFα. Subsequent studies [25], [27], however, failed to show any significant effect of TNFα on endothelial ANGPT2 expression or release. To verify the direct influence of TNFα on cellular ANGPT2 expression, we exposed both, human skeletal myoblasts and endothelial cells to TNFα for 24 h and found no significant changes in ANGPT2 expression in these cells (Figure 1). These results suggest that TNFα is unlikely to be the factor responsible for regulating satellite cell ANGPT2 expression.

There is increasing evidence that the production of angiogenesis factors by a variety of cells including skeletal muscles is under the control of reactive oxygen species (ROS). Indeed, several reports have confirmed that VEGF production by kertainocytes, skeletal myoblasts, endothelial and epithelial cells is stimulated by H_2_O_2_ [13], [28]–[30]. To evaluate whether ROS regulate ANGPT2 production in muscle and endothelial cells, we exposed human skeletal myoblasts and HUVECs to H_2_O_2_. Our study indicates that H_2_O_2_ elicits significant inductions of ANGPT2 production both in human skeletal myoblasts and in endothelial cells suggesting that both cell types can contribute to enhanced ANGPT2 production in response to oxidative stress (Figure 1). These results are in agreement with those of Amano *et al*., who reported elevated ANGPT2 expression in pericytes exposed to H_2_O_2_ [31]. The mechanisms, however, through which H_2_O_2_ induce ANGPT2 expression in skeletal muscles remain speculative. We speculate that H_2_O_2_ may activate ETS transcription factors [32] and that these factors, due to the abundance of their binding sites on human ANGPT2 promoter, are likely responsible for ANGPT2 production in both muscle and endothelial cells in response to oxidative stress [24].

### ANGPT2 regulation of myogenesis

The only available information regarding the influence of ANGPT2 on skeletal muscle is that of Dallabrida *et al.* [1], who reported that cultured skeletal satellite cells adhere to ANGPT1 and ANGPT2 proteins; however, whereas ANGPT1 promotes survival and inhibits apoptosis, ANGPT2 enhances apoptosis. We report here for the first time, that while ANGPT2 has no effect on proliferation and migration of skeletal myoblasts, it strongly promotes myoblast survival and inhibits apoptosis (Figure 2). ANGPT2 also strongly enhances differentiation of myoblasts into myotubes, as evidenced by substantial induction of muscle-specific transcription factors (MyoD and Myogenin) and muscle-specific proteins (Creatine Kinase and Myosin Heavy Chain) (Figure 4).

Differences between our conclusions and those of Dallabrida *et al*. regarding the influence of ANGPT2 on skeletal myoblast survival may be attributed to methodological differences. Dallabrida and colleagues focused their attention on measuring adhesion of myoblasts to extracellular matrices composed of ANGPT1, ANGPT2 or other proteins such as collagens, fibronectin, vitronectin and laminin. Their approach was based on an earlier report indicating that ANGPT1 is incorporated into extracellular matrices of cells [33]. However, it remains unknown whether or not ANGPT2 protein is actually incorporated into the extracellular matrices of skeletal myoblasts, so we assessed cell survival using a physiological approach that exposed skeletal myoblasts to ANGPT2 protein over 36 h.

We used two approaches to evaluate signaling mechanisms through which ANGPT2 regulates skeletal myoblast survival and differentiation. First, we measured activation of the PI3 kinase/AKT and ERK1/2 pathways in myoblasts exposed to ANGPT2. We found that ANGPT2 transiently and dose-dependently activates AKT and ERK1/2 phosphorylation and that ANGPT2 has no effect on starvation-induced cytotoxicity (index of overall cell death) and caspase 3/7 activation (index of apoptotic cell death) in the presence of selective inhibitors of PI3 kinase, AKT and ERK1/2. These results suggest that the PI3 kinase and ERK1/2 pathways are critical to the pro-survival and anti-apoptotic effects of ANGPT2 in skeletal muscle cells. This conclusion mirrors what has already been established in endothelial cells [34], although it is unclear in the case of skeletal myoblasts whether ANGPT2 pathway activation is mediated through phosphorylation of TIE2 receptors, ligation of integrins or both.

The second approach that we used to gain further insight into the mechanisms through which ANGPT2 modulates skeletal myoblast differentiation, survival and apoptosis was to compare the transcriptomes of myoblasts expressing GFP (control) and those overexpressing ANGPT2. Our results indicate that ANGPT2 triggers specific changes in the muscle transcriptome that involve the upregulation of several genes connected to cell signaling, including the regulation of cell cycle, assembly and organization, cell growth and proliferation. The protein that is most strongly-induced by ANGPT2 is NRP1 (neuropilin 1), which is a transmembrane receptor for semaphorins (mediators of neuronal guidance) and VEGFA [35]. NRP1 is expressed in skeletal muscle progenitor cells (myoblasts) and its expression is augmented during differentiation into myotubes [35]. These observations, along with the fact that VEGF strongly promotes skeletal myoblast survival and enhances myogenic differentiation, suggest that ANGPT2 may regulate myogenesis through augmentation of VEGF signaling. The protein that is second most induced by ANGPT2 is telomere maintenance 2 (TELO2), which functions as an S-phase checkpoint protein in the cell cycle and is a key element in embryonic development [36].

ANGPT2 also significantly induces the expression and release of leptin from myoblasts, suggesting that the pro-myogenic and pro-survival effects of ANGPT2 are likely mediated by leptin (figure 5). Leptin was identified initially as an adipocyte-derived hormone that regulates food intake through activation of receptors located in the hypothalamus. However, leptin is now recognized as a multipotent cytokine with numerous central and peripheral effects. Leptin and leptin receptor mRNA and proteins have been detected in mature skeletal muscle fibers and progenitor cells [37]. In muscle cells, leptin strongly influences metabolism by promoting both, free fatty acid oxidation and glucose uptake [38]. It also protects muscle cells from oxidative stress-induced apoptosis and prevents protein breakdown in C2C12 cultured myotubes [39], [40]. These observations suggest that the influence of ANGPT2 on skeletal muscle survival may be mediated through secondary release of leptin which can act on muscle cells through autocrine mechanism.

In addition to the above-described modulators of cell cycle and metabolism, network analysis of ANGPT2-regulated genes in muscle cells revealed that ANGPT2 downregulate many pro-inflammatory mediators including the inducible nitric oxide synthase, IL6 and members of cellular networks involving IFNα, IL1, IL12 and AP-1 and NFκB transcription factors (Figure 6 and Supporting Information S1). These cytokines and transcription factors are well known positive modulators of catabolism and muscle wasting. On the basis of these results, we speculate that ANGPT2 promotes muscle cells survival and differentiation through upregulation of key regulators of cell cycle, protein synthesis and metabolism and inhibition of the expression of mediators of protein degradation and catabolism.

In summary, our study indicates that ANGPT2 is expressed in primary human and skeletal myoblasts and that this expression is significantly induced upon differentiation of myoblasts to myotubes. We also found that skeletal ANGPT2 production is positively regulated by H_2_O_2_ and that ANGPT2 triggers significant increases in survival and differentiation of skeletal myoblasts. These effects of ANGPT2 are mediated through coordinated regulation of several modulators of cell survival, metabolism, protein synthesis and degradation.

## Supporting Information

Supporting Information S1 (DOC)
