# Supplementary material for: Expression and Functional Roles of Angiopoietin-2 in Skeletal Muscles
Source: PLoS One. 2011 Jul 29;6(7):e22882. doi: 10.1371/journal.pone.0022882 (PMC3146511; doi:10.1371/journal.pone.0022882)
Supplement: Supporting Information S1 — (DOC) [file pone.0022882.s001.doc]

**Supporting Information S1**

**Table S1:** Primers used for real-time PCR experiments to detect the expression of MyoD, Myogenin, Myosin Heavy Chain (MyoHC) and Creatine Kinase (CK) during differentiation of human skeletal myoblasts into myotubes.

**Table S2:** List of genes whose expression is significantly upregulated in skeletal myobalsts infected with Ad-ANGPT2 compared with cells infected with Ad-GFP.

**Table S3:** List of genes whose expression is significantly downregulated in skeletal myobalsts infected with Ad-ANGPT2 compared with cells infected with Ad-GFP.

**Table S4:** Primers used for real-time PCR experiments to detect the expression of TEL2, LEP, CSF3, ANGPTL4 and CTGF genes in human skeletal myoblasts infected with adenoviruses expressing GFP (Ad-GFP) and ANGPT2 (Ad-ANGPT2).


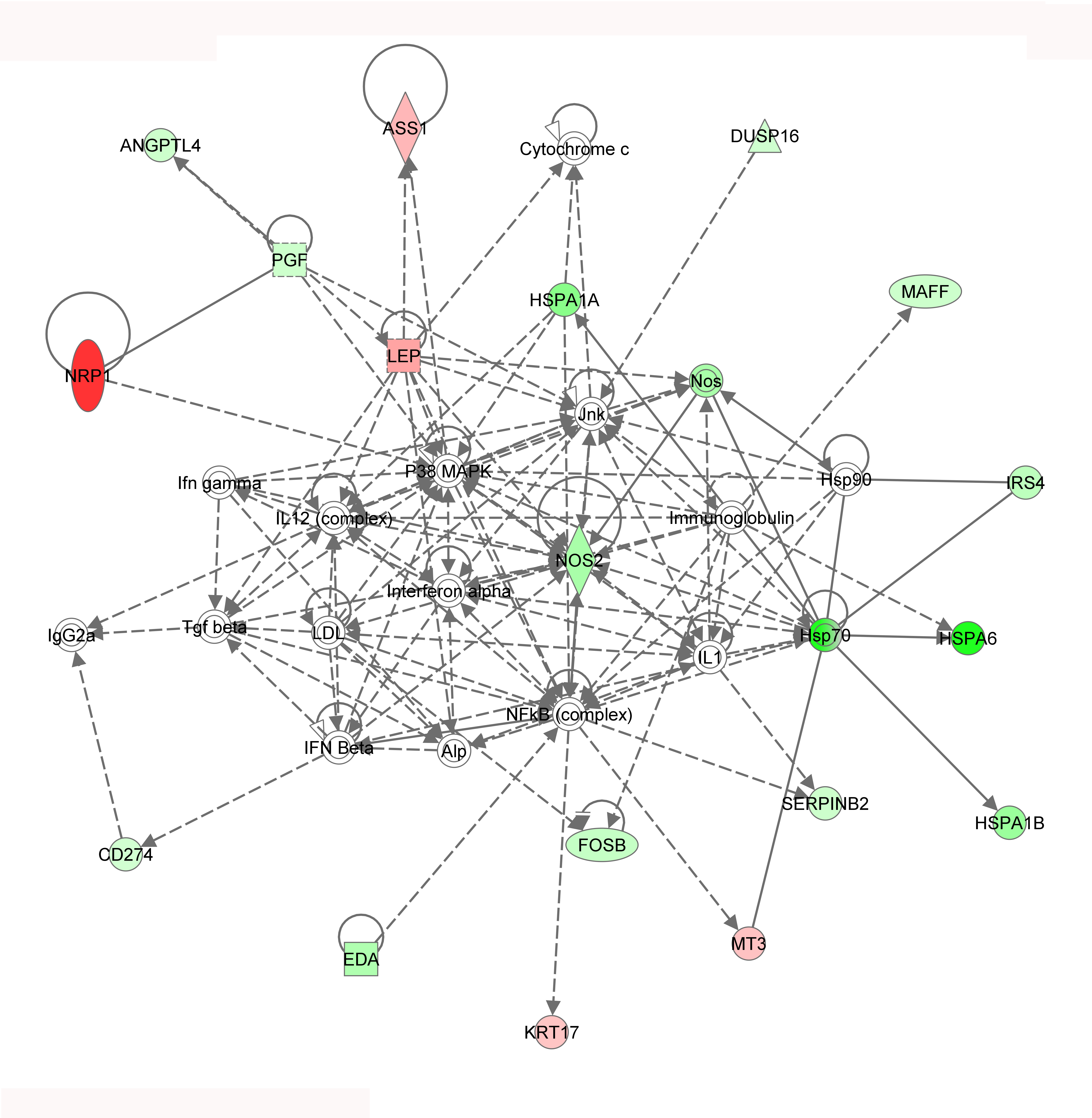


**Supplementary Figure 1:** The second top network of regulated genes in human myoblasts infected with Ad-ANGPT2 viruses vs. cells infected with Ad-GFP viruses (control condition). This figure was created with the Ingenuity Pathway Analysis system. Symbols are identical to those shown in supplementary figure 1. Top functions associated with this network include tissue morphology, lipid metabolism and small molecule biochemistry.
